# Synthesis and cytotoxic activities of novel copper and silver complexes of 1,3-diaryltriazene-substituted sulfonamides

**DOI:** 10.1080/14756366.2018.1530994

**Published:** 2018-10-26

**Authors:** Dilek Canakci, Ismail Koyuncu, Nabih Lolak, Mustafa Durgun, Suleyman Akocak, Claudiu T. Supuran

**Affiliations:** a Department of Chemistry, Vocational School of Technical Sciences, Adiyaman University, Adiyaman, Turkey;; b Faculty of Medicine, Department of Biochemistry, Harran University, Sanliurfa, Turkey;; c Faculty of Pharmacy, Department of Pharmaceutical Chemistry, Adiyaman University, Adiyaman, Turkey;; d Faculty of Arts and Sciences, Deparment of Chemistry, Harran University, Sanliurfa, Turkey;; e NEUROFARBA Department, Sezione di Scienze Farmaceutiche, Università degli Studi di Firenze, Florence, Italy

**Keywords:** Metal complexes, triazene, cancer cells, sulfonamides, cytotoxicity

## Abstract

In this study, a series of 10 novel copper (II) and silver complexes of 1,3-diaryltriazene-substituted sulfonamides was synthesised. All the synthesised ligands and their metal complexes were assessed for *in vitro* cytotoxicity against human colorectal adenocarcinoma (DLD-1), cervix carcinoma (HeLa), breast adenocarcinoma (MDA-MB-231), colon adenocarcinoma (HT-29), endometrial adenocarcinoma (ECC-1), prostate cancer (DU-145 and PC-3), normal embryonic kidney (HEK-293), normal prostate epithelium (PNT-1A), and normal retinal pigment epithelium (ARPE-19) cells. Most of the metal complexes from the series showed to be more active against all cancerous cells than the uncomplexed 1,3-diaryltriazene-substituted sulfonamides, and lower cytotoxic effects observed on normal cells. Most of the Cu (II) and Ag (I) metal complexes from the presented series showed high cytotoxic activity against HeLa cells with IC_50_ values ranging from 2.08 to >300 µM. Specifically, compound L_3_-Ag showed one of the highest cytotoxicity against all cancer cell lines with IC_50_ values between 3.30 to 16.18 µM among other tested compounds.

## 1. Introduction

Cancer is a group of the most fatal forms of diseases characterised by abnormal and uncontrolled cell proliferation. Cancer is the second most public cause of death after cardiovascular diseases across the world for men and women. On the other hand, incidence ratio is expected to increase dramatically in the near future. The high incidence and mortality ratio of cancer are due to the fact that there are more than 200 types of cancer and it is very hard to discover most of them in the early stage. For all these reasons, most majority of current research focused on cancer treatment with biologically more potent and less toxic way by using specific methods and techniques^1–3^.

Primary sulfonamides and their isoesters (sulfamides, sulfamates) constitute an important class of drugs with a wide variety of pharmacological applications^4–6^. Sulfonamides were also having an important place in drug discovery studies and continue to be the one of the most investigated compounds possessing different pharmacological activities such as, antibacterial, diuretic, anticarbonic anhydrase, protease inhibitory activity and more recently antitumor, among others^4–8^. Recently, one of the sulfonamide based CA inhibitor compound, which is ureido-substituted SLC-0111 was shown to be a highly effective hCA IX/XII inhibitor and reached to Phase I/II clinical trials for the treatment of advanced, metastatic breast cancer^9–11^.


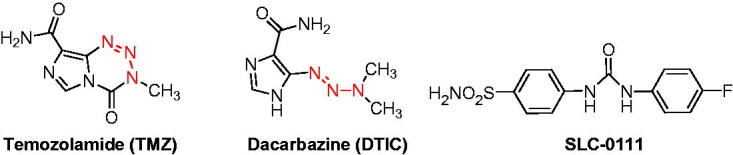


Triazenes (−N = N − NH−) are a diverse group of compounds which have been broadly investigated for synthetic reaction transformations and used for different applications such as natural product synthesis, combinatorial chemistry, and biomedical applications, among others^12^^,^^13^. The 1,3-diaryltriazene scaffold is one of the most interesting core owing to broad biological activities such as antibacteriel, antifungal, efficient carbonic anhydrase inhibitors, and their abundant use is in the development of novel anticancer molecules^12^^,^^13^. On the other hand, triazene compounds of clinical interest (such as Temozolamide and Dacarbazine), are a group of alkylating agents with excellent pharmacokinetic properties and limited toxicity^12^^,^^13^.

**Scheme 1.**
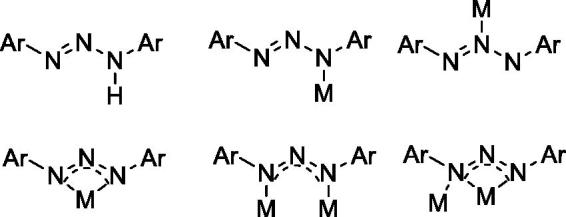
General binding modes of triazene ligands.

Triazene moieties can serve as monodentate binding through a terminal or central nitrogen, bidentate (N1, N3)-chelating to form bidentate complexes, and bridging ligands through (N1, N3)-bridging between two metal centers to from metallocycles over a wide variety of transition metal complexes^14^^,^^15^. Since metal-based drugs gained much attention after the discovery of cisplatin as an antitumor agent, 1,3-diaryltriazene based metal complexes started to be investigated more as a metallo-pharmaceuticals for several diseases especially for cancer (Scheme 1)^14^^,^^15^.

Generally, metals are essential components of cells chosen by nature^16^. They are frequently found in the enzyme catalytic domain^17^ and are involved in multiple biological processes, from the exchange of electrons to catalysis and structural roles^17^. They are extensively used in cellular activities. Such metals include gallium, zinc, cobalt, silver, vanadium, strontium, manganese and copper, which are required in trace amounts to trigger catalytic processes^18^. To this end, a balance between cellular need and the amount available in the body is important for the normal physiological state. Recently, there has been a growing demand for metal complex compounds in the treatment of cancer because of their improved biological activity than their corresponding free ligands^19^.

It has been well accepted that hybrid molecules through the combination of different scaffolds and pharmacophores into a single compound may lead to the improved cytotoxic potency of compounds with synergistic effect. According to literature knowledge and our previous studies^13–15^^,^^20^, in the current work, novel copper (II) and silver complexes incorporating 1,3-diaryltriazene-substituted sulfonamides were synthesised in attempt to obtain possible active compounds having cytotoxic activities against DLD-1, HeLa, MDA-MB-231, HT-29, ECC-1, DU-145, PC-3, HEK-293, PNT-1A, and ARPE-19 cancer cell lines. Our aim was to investigate new metal complexes to have a potent cytotoxic effect with a low toxicity.

## 2. Materials and methods

### 2.1. General

All chemicals and anhydrous solvents were purchased from Sigma-Aldrich, Merck, Alfa Aesar and TCI and used without further purification. Melting points (mp) were determined with SMP30 melting point apparatus in open capillaries and are uncorrected. FT-IR spectra were recorded by using Perkin Elmer Spectrum 100 FT-IR spectrometer. Ultraviolet–visible (UV–vis) absorption spectra were recorded on Shimadzu UV-2101 spectrophotometer in DMSO. Nuclear Magnetic Resonance (^1^H-NMR and 13C-NMR) spectra of compounds were recorded using a Bruker Advance III 300 MHz spectrometer in DMSO-d_6_ and TMS as an internal standard operating at 300 MHz for ^1^H-NMR and 75 MHz for ^13^C-NMR. Thin layer chromatography (TLC) was carried out on Merck silica gel 60 F_254_ plates.

The ligands 3–(3-(4-fluorophenyl)triaz-1-en-1-yl) benzenesulfonamide (**L_1_**), 3–(3-(4-methoxyphenyl)triaz-1-en-1-yl) (**L_2_**), benzenesulfonamide 4–(3–(3-sulfamoylphenyl)triaz-2-en-1-yl) benzoic acide (**L_3_**), 3–(3-(3,4-dimethoxyphenyl)triaz-1-en-1-yl) benzenesulfonamide (**L_4_**), and 3-(3-(3,5-dimethylphenyl)triaz-1-en-1-yl)benzenesulfonamide (**L_5_**) were synthesised and characterised as previously described by us^20^.

### 2.2. General procedure for the preparation of coordination compounds (L_1-5_ Cu)

The copper complexes of 1,3-diaryltriazene-substituted metanilamides were prepared using chemical precipitation of ion Cu^2+^ with a molar ratio of 1:1. Briefly, Copper(II) acetate monohydrate (1.0 mmol) was dissolved in ethanol (10 ml) at room temperature. The ligands (**L_1-5_**) (1.0 mmol) dissolved in ethanol (10 ml) were added into resulting solution drop by drop. The mixture was refluxed for 3 h and after cooling, the resulting solution was partially evaporated. The precipitate was separated by filtration, washed with 1:1 (v/v ethanol/water) and the pure complexes were dried in a desiccator over anhydrous calcium chloride at room temperature.

#### 2.2.1. L_1_-Cu

Yield: 41%; Color: brown solid; mp: >300 ^0^C; UV–Vis (DMSO, nm): 232, 261, 305, 359, 460; FT-IR (cm^−1^): 3374, 3077 (NH_2_), 1666 (C=C), 1437 (N=N), 1330 (asymmetric), 1162 (symmetric) (S=O); ^1^H-NMR (DMSO-d_6_, 300 MHz, δ ppm): 7. 92 (s, 1H, Ar-H), 7.70–7.42 (m, 5H, Ar-H), 7.49 (s, 2H, −SO_2_NH_2_), 7.32 (t, 2H, *J* = 3.3, Ar-H): 13C-NMR (DMSO-d_6_, 75 MHz, δ ppm): 158.6, 151.9, 145.6, 138.2, 130.4, 124.8, 123.5, 119.8, 116.6, 115.7;

#### 2.2.2. L_2_-Cu

Yield: 45%; Color: brown solid; mp: >300 ^0^C; UV–Vis (DMSO, nm): 239, 254, 303, 409, 461; FT-IR (cm^−1^): 3256, 3000 (NH_2_), 1586 (C=C), 1457 (N=N), 1320 (asymmetric), 1155 (symmetric) (S=O); ^1^H-NMR (DMSO-d_6_, 300 MHz, δ ppm): 7. 95 (s, 1H, Ar-H), 7.85–7.78 (m, 5H, Ar-H), 7.75–7.68 (m, 2H, Ar-H), 7.54 (s, 2H, −SO_2_NH_2_), 3.86 (s, 3H, −OCH_3_): 13C-NMR (DMSO-d_6_, 75 MHz, δ ppm): 159.4, 151.2, 145.9, 138.4, 130.2, 124.5, 123.7, 119.9, 116.4, 115.3, 55.6;

#### 2.2.3. L_3_-Cu

Yield: 38%; Color: brown solid; mp: >300 ^0^C; UV–Vis (DMSO, nm): 245, 255, 278, 364, 440; FT-IR (cm^−1^): 3570, 3075 (NH_2_), 1591 (C=C), 1471, 1330 (asymmetric), 1124 (symmetric) (S=O); ^1^H-NMR (DMSO-d_6_, 300 MHz, δ ppm): 12.92 (br.s, 1H, −COOH), 7. 90 (s, 1H, Ar-H), 7.75–7.58 (m, 5H, Ar-H), 7.50 (s, 2H, −SO_2_NH_2_), 7.45–7.33 (m, 2H, Ar-H): 13C-NMR (DMSO-d_6_, 75 MHz, δ ppm): 179.5, 159.1, 151.7, 145.9, 138.8, 130.7, 124.2, 123.5, 119.7, 116.8, 115.6;

#### 2.2.4. L_4_-Cu

Yield: 43%; Color: brown solid; mp: >300 ^0^C; UV–Vis (DMSO, nm): 233, 246, 260, 309, 363, 453; FT-IR (cm^−1^): 3412, 2956 (NH_2_), 1627 (C=C), 1415 (N=N), 1350 (asymmetric), 1110 (symmetric) (S=O); ^1^H-NMR (DMSO-d_6_, 300 MHz, δ ppm): 8.25 (s, 1H, Ar-H), 8.15–8.09 (m, 1H, Ar-H), 7.73–7.61 (m, 3H, Ar-H), 7.49 (s, 2H, −SO_2_NH_2_), 7.39 (s, 1H, Ar-H), 6.45 (s, 1H, Ar-H), 3.85 (s, 3H, −OCH_3_), 3.80 (s, 3H, −OCH_3_),: 13C-NMR (DMSO-d_6_, 75 MHz, δ ppm): 160.5, 151.9, 146.3, 139.7, 138.5, 131.2, 130.6, 125.7, 123.6, 119.1, 116.6, 115.3, 55.9, 55.5;

#### 2.2.5. L_5_-Cu

Yield: 37%; Color: brown solid; mp: >300 °C; UV–Vis (DMSO, nm): 226, 279, 310, 361, 524; FT-IR (cm^−1^): 3341, 3080 (NH_2_), 1598 (C=C), 1484 (N=N), 1310 (asymmetric), 1124 (symmetric) (S=O); ^1^H-NMR (DMSO-d_6_, 300 MHz, δ ppm): 8.10 (s, 1H, Ar-H), 7.92–7.68 (m, 2H, Ar-H), 7.70–7.64 (m, 2H, Ar-H), 7.50 (s, 2H, −SO_2_NH_2_), 6.48 (s, 2H, Ar-H), 2.52 (s, 6H, −CH_3_): 13C-NMR (DMSO-d_6_, 75 MHz, δ ppm): 160.1, 151.5, 146.8, 139.2, 131.1, 125.7, 123.4, 119.5, 116.1, 115.3, 21.5;

### 2.3. General procedure for the preparation of coordination compounds (L_1-5_ Ag)

Ethanol (20 ml) and silver acetate (1 mmol) were taken into boiling flask and stirred for 30 min at room temperature. At the end of the period, **L_1−5_** (1 mmol) dissolved in ethanol (10 ml) was added drop by drop into solution. The mixture was thoroughly mixed and allowed to reflux for 3 h. The resulting solution was partially evaporated. The precipitate was separated by filtration, washed with 1:1 (v/v ethanol/water) and the pure complexes were dried in a desiccator over anhydrous calcium chloride at room temperature.

#### 2.3.1. L_1_-Ag

Yield: 36%; Color: light brown solid; mp: >300 °C; UV–Vis (DMSO, nm): 259, 288, 413, 454; FT-IR (cm^−1^): 3420, 3069 (NH_2_), 1596 (C=C), 1430 (N=N), 1332 (asymmetric), 1157 (symmetric) (S=O); ^1^H-NMR (DMSO-d_6_, 300 MHz, δ ppm): 7. 90 (s, 1H, Ar-H), 7.72–7.43 (m, 5H, Ar-H), 7.48 (s, 2H, −SO_2_NH_2_), 7.30 (t, 2H, *J* = 3.3, Ar-H): ^13^C-NMR (DMSO-d_6_, 75 MHz, δ ppm): 158.9, 151.3, 145.7, 138.1, 130.3, 124.3, 123.8, 119.1, 116.7, 115.4;

#### 2.3.2. L_2_-Ag

Yield: 25%; Color: light brown solid; mp: >300 °C; UV–Vis (DMSO, nm): 247, 294; FT-IR (cm^−1^): 3234, 3000 (NH_2_), 1580 (C=C), 1431 (N=N), 1325 (asymmetric), 1153 (symmetric) (S=O); ^1^H-NMR (DMSO-d_6_, 300 MHz, δ ppm): 7. 93 (s, 1H, Ar-H), 7.84–7.75 (m, 5H, Ar-H), 7.74–7.66 (m, 2H, Ar-H), 7.51 (s, 2H, −SO_2_NH_2_), 3.85 (s, 3H, −OCH_3_): ^13^C-NMR (DMSO-d_6_, 75 MHz, δ ppm): 159.9, 151.4, 145.5, 138.2, 130.6, 124.7, 123.2, 119.5, 116.3, 115.1, 55.5;

#### 2.3.3. L_3_-Ag

Yield: 36%; Color: light brown solid; mp: >300 °C; UV–Vis (DMSO, nm): 268, 276, 312, 359, 423; FT-IR (cm^−1^): 3229, 3065 (NH_2_), 1690 (C=O), 1602 (C=C), 1427 (N=N), 1334 (asymmetric), 1159 (symmetric) (S=O); ^1^H-NMR (DMSO-d_6_, 300 MHz, δ ppm): 12.90 (br.s, 1H, −COOH), 7. 88 (s, 1H, Ar-H), 7.73–7.59 (m, 5H, Ar-H), 7.49 (s, 2H, −SO_2_NH_2_), 7.43–7.34 (m, 2H, Ar-H): ^13^C-NMR (DMSO-d_6_, 75 MHz, δ ppm): 179.2, 159.4, 151.5, 145.6, 138.2, 130.5, 124.6, 123.4, 119.5, 116.9, 115.3;

#### 2.3.4. L_4_-Ag

Yield: 36%; Color: light brown solid; mp: >300 °C; UV–Vis (DMSO, nm): 259, 288, 413, 454; FT-IR (cm^−1^): 3420, 3069 (NH_2_), 1596 (C=C), 1430 (N=N), 1345 (asymmetric), 1157 (symmetric) (S=O); ^1^H-NMR (DMSO-d_6_, 300 MHz, δ ppm): 8.22 (s, 1H, Ar-H), 8.11–8.05 (m, 1H, Ar-H), 7.70–7.60 (m, 3H, Ar-H), 7.48 (s, 2H, −SO_2_NH_2_), 7.37 (s, 1H, Ar-H), 6.44 (s, 1H, Ar-H), 3.83 (s, 3H, −OCH_3_), 3.79 (s, 3H, −OCH_3_),: ^13^C-NMR (DMSO-d_6_, 75 MHz, δ ppm): 160.3, 151.7, 146.5, 139.5, 138.1, 131.6, 130.7, 125.3, 123.2, 119.4, 116.1, 115.4, 55.8, 55.4;

#### 2.3.5. L_5_-Ag

Yield: 32%; Color: light brown solid; mp: >300 °C; UV–Vis (DMSO, nm): 231, 250, 293, 531; FT-IR (cm^−1^): 3368, 3255 (NH_2_), 1589 (C=C), 1434 (N=N), 1320 (asymmetric), 1163 (symmetric) (S=O); ^1^H-NMR (DMSO-d_6_, 300 MHz, δ ppm): 8.09 (s, 1H, Ar-H), 7.90–7.69 (m, 2H, Ar-H), 7.65–7.60 (m, 2H, Ar-H), 7.48 (s, 2H, −SO_2_NH_2_), 6.45 (s, 2H, Ar-H), 2.50 (s, 6H, −CH_3_): ^13^C-NMR (DMSO-d_6_, 75 MHz, δ ppm): 160.0, 151.4, 146.5, 139.1, 131.0, 125.4, 123.2, 119.2, 116.5, 115.8, 21.6;

### 2.4. *In vitro* cytotoxic activity

#### 2.4.1. Cell cultures

The cells studied were obtained from the American Type Culture Collection (ATCC, Manassas, VA), and included human colorectal adenocarcinoma (DLD-1), cervix carcinoma (HeLa), breast adenocarcinoma (MDA-MB-231), colon adenocarcinoma (HT-29), endometrial adenocarcinoma (ECC-1), prostate cancer (DU-145 and PC-3), normal embryonic kidney (HEK-293), normal prostate epithelium (PNT-1A), and normal retinal pigment epithelium (ARPE-19) cells. As recommended by ATCC, cells were subjected to propagation using DMEM-F12 and RPMI-1640 media, with supplements 10% fetal bovine serum, L-glutamine (2 mM), penicillin (100 U/mL) and streptomycin (100 mg/mL) in a humidified atmosphere (5% CO_2_) at 37 °C. When the cultures reached 70–80% confluence, the cells were harvested using 0.25% trypsin (Sigma).

#### 2.4.2. Cytotoxicity assays and determination of IC_50_

Cytotoxic activities were based on the reduction of (3–(4,5-dimethylthiazol-2-yl)-2,5-diphenyltetrazolium bromide) (MTT) by mitochondrial dehydrogenase of viable cells to give a blue formazan product which can be measured spectrophotometrically by UV–Vis measurements. MTT colorimetric assays were performed using 96 well plates. The cells were seeded in a 96 well plate at concentration 5.0 × 10^4^ cells/well and incubated at 37 °C for 24 h. After treatment with various concentrations of the test compound (1, 5, 10, 25, 50, 100 and 200 μM), the cells were incubated for an additional 48 h at 37 °C. After incubation, the medium was removed, and the cells in each well were incubated with 100 μL MTT solution (5 mg/mL) for 4 h at 37 °C. MTT solutions were then discarded, and 100 μL DMSO were added to dissolve insoluble formazan crystals. Optical densities were measured at 570 nm (Varioskan Flash Multimode Reader, Thermo, Waltham, MA). Data were obtained from triplicate wells. Cytotoxic effects were determined in reference to negative controls (vehicle treated cells). Cytotoxicity was expressed as the mean percentage increase relative to the unexposed control (mean ± SD). All statistical analyses were performed using SPSS package program for Windows (Version 20, Chicago, IL). Control values were set to 0% cytotoxicity. Cytotoxicity data, where appropriate, were fitted to sigmoidal curves and a four-parameter logistic model was used to calculate the IC_50_ values, the concentration of material causing 50% inhibition, compared to the untreated controls. 5-Fluorouracil (5-FU) was also used as a control agent^21^^,^^22^.

## 3. Results and discussion

### 3.1. Synthesis and characterisation of the metal complexes

**Scheme 2.**
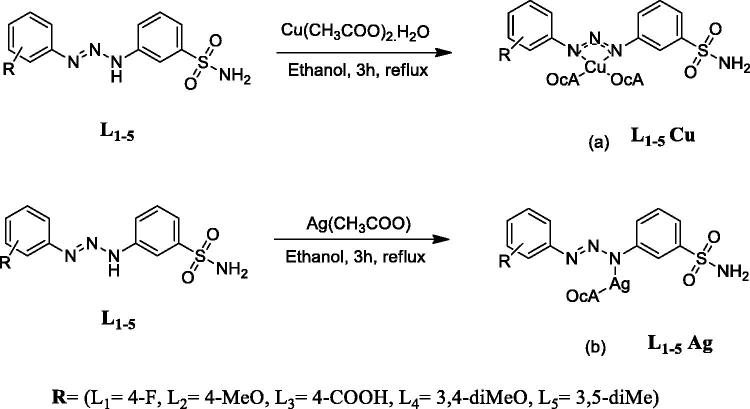
General synthetic route of the novel copper (a) and silver (b) complexes.

The copper (II) and silver complexes were synthesised from the reaction of the appropriate 1,3-diaryltriazene-substituted sulfonamide ligands and metal salt. The structures of Cu and Ag 1,3-diaryltriazene metal complexes (Cu^+2^ and Ag^+^) were elucidated by ^1^H-NMR, ^13^C-NMR, UV–Vis and FT-IR spectroscopic analysis. All spectral data were in agreement with calculated values of proposed structures (Scheme 2).

FT-IR spectra of all synthesised 1,3-diaryltriazene-substituted sulfonamides and metal complexes were recorded in the range of 450–4000 cm^−1^. The FT-IR spectrum of ligands shows bands around 3350 and 3000 cm^−1^. These bands resulted from stretching vibrations of protons (N–H) in the structure of triazene and sulfonamide. The band forming around 1600 cm^−1^, on the other hand, represents ν(N=N) stretching vibration. The ν(N=N) stretching vibration band formed in the region of ν(C=O) stretching band because of the tautomeric forms of the compound. Another reason was ν(N-H) plane and out-of- plane bending vibrations. The peaks in the range of 1100–1380 cm^−1^ were attributes to symmetric and assymmetric the ν(S=O) stretching frequency. Aromatic C–H stretching vibration bands were observed in the range of 2916–3017 cm^−1^. Bands in the range of 1400–1590 cm^−1^ occurred as a result of aromatic ν(C=C) and ν(N=N) stretching vibrations.

According to spectra of complexes, the most significant difference with ligand spectrum was in the range of 2900–3600 cm^−1^. ν(N-H) bands which were considerably sharp in triazene gained a broad appearance as a result of the formation of complex. The reason for this was that formation of complex took place through metal and nitrogen atoms. Bands seen at around 3350 and 3250 cm^−1^ in the spectrum of metal complexes belonged to ν(N–H) stretching vibration. Bands forming at around 2900 and 3000 cm^−1^ occurred as a result of aromatic ν(C–H) stretching vibration. In addition, intensity of bands and the number of bands decreased as a result of complex formation. Aromatic ν(C–H) vibration band was not observed in the spectrum because it overlapped with ν(N–H) stretching vibration bands. While ν(N=N) stretching vibration band formed around 1590 cm^−1^, aromatic ν(C=C) and ν(N–N) stretching vibration bands occurred at around 1550 and 1450 cm^−1^, respectively. Another feature supporting the formation of complex was that some peaks were not seen in spectra of finger print site. The bands due to the metal-ligand stretching modes are expected to be present in the low frequency region between 650 and 200 cm^−1^.

The UV–Vis absorption spectra of all 1,3-diaryltriazenes and metal complexes with Cu^+2^ and Ag^+^ were measured in DMSO in a concentration of 10^−5 ^mol L^−1^ in the wavelength range of 200–700 nm. Table 1 shows electronic absorption spectra of triazene ligands and cooper and silver complexes. Although the main structure of all the triazene compounds is similar, different shifts are seen in the UV–Vis spectrum due to the fact that the groups bound to the benzene ring are different. All compounds showed almost identical absorption maxima in the ultraviolet region in the range of 200–260 nm, corresponding to π→π* transitions of benzenoid system of the compounds. The band observed in the range 273–312 nm is assigned to low energy π→π* electronic transition of triazene and phenly rings. The high intensity n→π* band in the range of 341–367 nm occurred due to the conjugation between aromatic ring system and triazene group. The band in the range of 401–531 nm can be regarded as n–π* electronic transition involving the whole electron system with charge transfer interaction within the molecules. The UV absorption band of **L_5_** in visible region was found to be the highest among all triazene compounds. This is because of the reason that the compound has electron donating groups which causes bathochromic shift to 496 nm. Table 1 shows absorption maxima of all compounds in the visible region and the charge transfer energies (*E*_CT_ = 1243,667/λ_CT_) calculated from wavelengths of absorption maxima. When wavelengths of ligands and complexes at visible region were compared, the highest value belonged to **L_5_** from ligands and **L_4_-Ag** from complexes. As an electron-transfer trend increases, less energy is required for charge-transfer process and the resulting complex has absorption at greater wavelengths. Wavelengths of silver complexes were higher compared to copper complexes. Low energy bands occurring in 411–461 nm region at electronic spectra of copper (II) complexes and in the range of 423–531 nm in the spectra of silver (I) complex occurred as a result of d-d and metal-to-ligand charge transfers.

**Table 1.** The UV–Vis spectra data of 1,3-diaryltriazene ligands and metal complexes.

	A		B		C		D		
Compounds	*λ*_max_	*ε*_max_ × 10^−5^	*λ*_max_	*ε*_max_ × 10^−5^	*λ*_max_	*ε*_max_ × 10^−5^	*λ*_max_	*ε*_max_ × 10^−5^	*E*_CT_
L_1_	233	0.389	309	3.289	360	3.148	401	2.637	3.133
L_2_	260	3.585	289	3.937	366	2.652	420	2.792	2.961
L_3_	254	1.629	303	3.547	360	3.323	416	2.949	2.990
L_4_	254	1.561	273	0.486	341	1.500	496	1.442	2.507
L_5_	255	2.881	294	3.730	341	1.503	497	3.374	2.502
L_1_-Cu	262	3.850	288	4.663	353	2.914	427	2.641	2.913
L_2_-Cu	254	2.294	303	3.645	360	3.399	461	4.364	2.698
L_3_-Cu	234	3.842	281	5.273	361	3.113	422	2.819	2.947
L_4_-Cu	260	1.448	309	3.142	363	3.020	453	3.145	2.745
L_5_-Cu	222	4.383	279	6.561	366	2.474	411	2.581	3.026
L_1_-Ag	248	4.537	287	5.096	–	–	438	4.104	2.839
L_2_-Ag	247	1.996	285	1.913	–	–	447	3.286	2.782
L_3_-Ag	268	3.483	312	3.529	359	3.332	423	3.177	2.940
L_4_-Ag	256	2.154	293	3.676	–	–	531	3.373	2.342
L_5_-Ag	259	2.846	292	2.540	367	2.857	480	3.509	2.591

*λ*_max_: the wavelength at the absorption maxima(nm); *ε*_max_: molar absoptivity coefficient(L/mol cm); *E*_CT_: charge transfer energy(eV).

^1^H-NMR and 13C-NMR spectra of all 1,3-diaryltriazene and metal complexes were recorded in DMSO-d_6_ relative to TMS as reference. Peaks of aromatic hydrogen in the spectra of copper and silver complexes occurred in regions that were similar to their ligands. The most distinct difference in spectra of ligands and complexes was the absence of triazene hydrogen peaks in complex spectra. The − NH − peak was observed around 12.75 ppm in the ligands, but there was no peak around 12.75 ppm in the metal complexes. This was an obvious evidence for that formation of the complex took place through nitrogen atoms of triazene. There is no significant change in peak positions corresponding to the sulfonamide protons in the complexes.

### 3.2. Cytotoxicity studies

In order to investigate the cytotoxic efficiency of novel copper (II) and silver 1,3-diaryltriazene-substituted metanilamide complexes on seven cancer cell lines together with three normal cell lines, MTT assay was performed. The cancer cells included human colorectal adenocarcinoma (DLD-1), cervix carcinoma (HeLa), breast adenocarcinoma (MDA-MB-231), colon adenocarcinoma (HT-29), endometrial adenocarcinoma (ECC-1), prostate cancer (DU-145 and PC-3), and the normal cells used were embryonic kidney (HEK-293), prostate epithelium (PNT-1A), and retinal pigment epithelium (ARPE-19) cells. In the Table 2, IC_50_ values (concentration required to inhibit tumor cell proliferation by 50%) of newly prepared Cu and Ag metal complexes of 1,3-diaryltriazene derivatives were summarised.

**Table 2.** Cytotoxicity of ligands L_1-5_, metal complexes of L_1-5_ Cu, L_1-5_ Ag and 5-fluorouracil (5-FU) on tumor cell lines and normal cell lines.

Compound	IC_50_ (µM)^a^^,b^									
	Cancer cell							Normal cell		
	DLD-1	HeLa	MDA-MB-231	HT-29	ECC-1	DU-145	PC-3	HEK-293	PNT-1A	ARPE-19
L_1_	139.8 ± 11.0	178.8 ± 16	104.1 ± 28.2	79.4 ± 15.4	239.4 ± 27.5	29.0 ± 2.9	168.2 ± 18.5	131.7 ± 16.4	194.9 ± 23.5	279.2 ± 33.8
L_2_	223.0 ± 8.3	768 ± 6.5	144.4 ± 2.4	265.2 ± 30	>300	154.4 ± 25.6	170.6 ± 20.5	145.7 ± 13.4	>300	>300
L_3_	>300	>300	>300	>300	197.0 ± 23.5	186.5 ± 20.6	>300	>300	>300	>300
L_4_	>300	>300	155.1 ± 2.6	>300	>300	181.9 ± 23.4	299.4 ± 32.3	>300	237.1 ± 26.8	>300
L_5_	223.6 ± 12.4	65.1 ± 4.5	144.1 ± 18.6	111.6 ± 16.5	131.6 ± 13.1	97.8 ± 9.7	131.0 ± 12.4	102.7 ± 10.2	177.4 ± 20.5	>300
L_1_-Cu	47.1 ± 4.7	5.0 ± 0.4	116.8 ± 14.2	198.9 ± 24.3	26.2 ± 2.6	104.9 ± 13.5	102.0 ± 10.2	68.8 ± 8.7	76.5 ± 8.5	119.0 ± 13.5
L_2_-Cu	42.7 ± 3.3	2.1 ± 0.1	50.0 ± 2.4	94.9 ± 9.4	39.5 ± 2.5	116.4 ± 14.6	65.3 ± 8.5	159.2 ± 19.4	95.1 ± 11.1	131.7 ± 15.6
L_3_-Cu	65.2 ± 2.5	63.9 ± 4.3	48.3 ± 2.5	205.1 ± 20.3	362.1 ± 38.1	58.3 ± 6.4	196.4 ± 22.6	134.7 ± 15.6	119.5 ± 13.5	>300
L_4_-Cu	58.4 ± 5.8	115.8 ± 9.2	205.5 ± 25.1	198.7 ± 22.5	307.5 ± 34.2	160.5 ± 18.0	296.8 ± 32.4	196.8 ± 23.5	125.1 ± 14.8	105.2 ± 13.5
L_5_-Cu	58.8 ± 3.9	15.2 ± 1.0	26.0 ± 2.5	84.4 ± 8.4	97.6 ± 9.7	32.4 ± 3.2	79.2 ± 8.5	36.3 ± 3.6	46.4 ± 4.6	84.3 ± 8.4
L_1_-Ag	279.3 ± 12.9	>300	96.8 ± 9.6	185.7 ± 16.5	207.0 ± 25.6	136.7 ± 16.5	612.0 ± 61.2	>300	209.7 ± 20.9	>300
L_2_-Ag	31.6 ± 3.2	2.8 ± 0.1	30.3 ± 3.0	24.9 ± 2.5	29.1 ± 3.0	18.6 ± 1.8	28.2 ± 3.5	17.8 ± 1.7	17.5 ± 17.5	22.0 ± 10.2
L_3_-Ag	3.3 ± 0.3	3.4 ± 0.2	9.8 ± 0.9	10.1 ± 3.1	16.2 ± 1.6	4.8 ± 0.4	5.1 ± 0.5	9.9 ± 0.9	9.2 ± 0.9	9.5 ± 0.9
L_4_-Ag	173.3 ± 10.3	139.3 ± 28	>300	240.7 ± 28.5	103.6 ± 10.3	236.7 ± 28.5	136.6 ± 15.8	5.5 ± 0.6	139.1 ± 16.5	284.8 ± 30.5
L_5_-Ag	110.7 ± 9.0	9.6 ± 0.6	148.4 ± 19.5	161.6 ± 16.1	135.9 ± 13.5	100.3 ± 10.1	111.7 ± 13.4	30.5 ± 4.5	110.8 ± 11.0	109.4 ± 11.0
5-FU	50.2 ± 21.0	19.2 ± 1.2	22.4 ± 2.5	24.2 ± 2.4	30.6 ± 3.5	37.3 ± 5.8	45.5 ± 4.5	65.3 ± 8.6	142.3 ± 18.6	75.3 ± 7.5

a Values are means of three independent experiments.

b IC_50_ values determined at 48 h.

**Figure 1.**
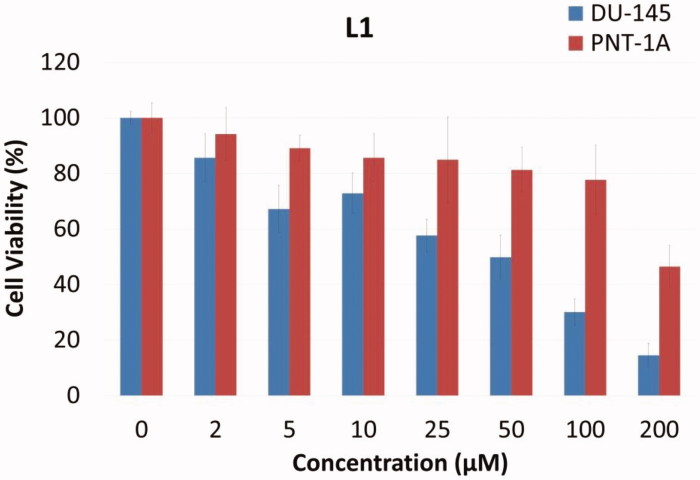
Plot of viable cells at various concentrations of L_1_ against DU-145 tumor cells and PNT-1A normal cell line.

In general, from Table 2, it can be seen that Cu (II) and Ag (I) complexes of 1,3-diaryltriazene derivatives showed more cytotoxic efficiency than their ligands. Specifically, the most efficient cytotoxic effect was observed against HeLa cancer cell line with metal complexes of L_1_-Cu, L_2_-Cu, L_2_-Ag, L_3_-Ag, and L_5_-Ag with IC_50_ values of 5.02, 2.08, 2.80, 3.40, and 9.61 µM, respectively. Among the series, the metal complex L_3_-Ag showed greater cytotoxicity against all cancer cell lines with IC_50_ values ranging from 3.30 to 16.18 µM. The compound L_2_-Ag was also more effective against most of the cancer cell lines as compared to 5-FU with IC_50_ values of 31.56 (DLD-1), 2.80 (HeLa), 29.11 (ECC-1), 18.63 (DU-145) and 28.18 (PC-3) µM. Among the uncomplexed ligands, the most effective cytotoxicity was observed against DU-145 cancer cell line with IC_50_ values in the range of 18.96 to 186.56 µM. More specifically, the ligand L_1_ showed highest cytotoxicity against prostate cancer (DU-145) (IC_50_: 28.96 M), but, on the other hand, against normal prostate cells (PNT1-A) the cytotoxicity was much lower (IC_50_: 194.89 M) (Figure 1). According to these results, the ligand L_1_ possessed high selectivity between prostate cancer cell line (DU-145) and normal prostate cells (PNT1-A).

**Figure 2.**
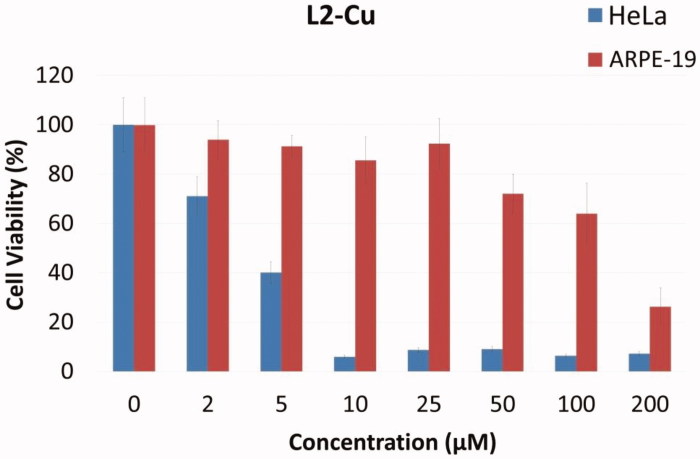
Plot of viable cells at various concentrations of L2-Cu against HeLa tumor cells and ARPE-19 normal cell line.

Among the Cu (II) complexes, the L_2_-Cu showed great cytotoxic activity against all cancer cell lines that were assessed in the this work. Regarding activity against the highest metastised effective HeLa cancer cell line, the L_2_-Cu emerged as the most active one that displayed cytotoxic activity with IC_50_ value equals to 2.08 µM, which is comparable to the reference drug 5-FU (IC_50_: 19.15 µM) (Figure 2).

**Figure 3.**
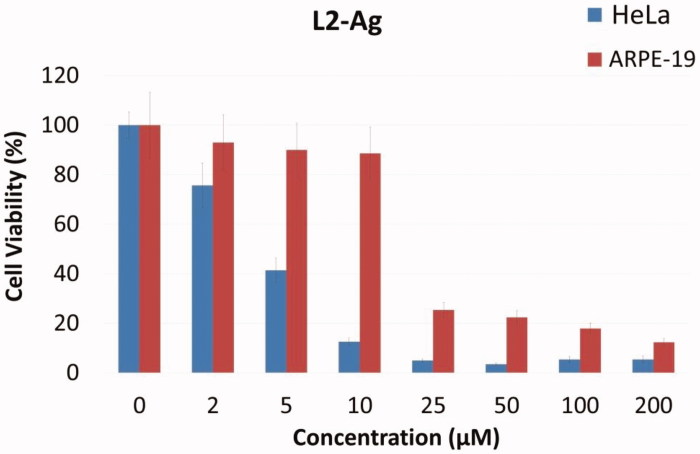
Plot of viable cells at various concentrations of L_2_-Ag against HeLa tumor cells and ARPE-19 normal cell line.

On the other hand, investigation of the cytotoxicity towards the cervix carcinoma HeLa cells elucidated that L_2_-Ag complex had the highest cytotoxicity (IC_50_: 2.80 µM) among the Ag (I) complexes, with 6.84 fold increased potency than the reference drug 5-FU (IC_50_: 19.15 µM) (Figure 3).

## 4. Conclusion

We investigated a series of copper (II) and silver (I) complexes of 1,3-diaryltriazene-substituted sulfonamide derivatives. The cytotoxic activity of these ligands and their Cu (II) and Ag (I) complexes were evaluated against seven cancer cell lines (DLD-1, HeLa, MDA-MB-231, HT-29, ECC-1, DU-145, PC-3) as well as three normal cell lines (HEK-293, PNT-1A, and ARPE-19). Most of the metal complexes showed better cytotoxic potency than their ligands and comparable potency to that of 5-Fluorouracil (5-FU), which is a commonly used drug. Specifically, one of the metal complexes (**L_3_-Ag**) from the series presented great cytotoxic activity against all cancer cell lines that were tested in this study with IC_50_ values in the range of 3.30–16.18 µM. As a result, this work encouraged us to investigate their anticancer profiles in further studies.
